# Real-time analysis of ATP concentration in acupoints during acupuncture: a new technique combining microdialysis with patch clamp

**DOI:** 10.1186/s13036-019-0221-0

**Published:** 2019-11-28

**Authors:** Yong Wu, Meng Huang, Ying Xia, Guanghong Ding

**Affiliations:** 1 Department of Aeronautics and Astronautics, Fudan University, No.220, Rd. Handan, Shanghai, 200433 China; 2 Shanghai Key Laboratory of Acupuncture Mechanism and Acupoint Function, Fudan University, No.220, Rd. Handan, Shanghai, 200433 China

**Keywords:** Microdialysis, Patch clamp, ATP, Online real-time analysis, Acupoint

## Abstract

This paper introduces a new technique combining microdialysis with patch clamp to detect the changes in ATP (adenosine triphosphate) concentration in acupoints during acupuncture. The microdialysis probe was implanted into the Zusanli acupoint (ST 36) of adult SD (Sprague Dawley) rats to sample acupoint fluid containing ATP released during acupuncture. Then, the fluid with ATP was delivered in real time to 293 T cells overexpressing P2X3 receptors, with which we could carry out patch clamp experiments. The results showed that changes in membrane currents could reflect changes in the concentration of ATP. Thus, we can successfully detect ATP released in acupoints during acupuncture in real time. This technique provides us with a new way to study the mechanism of acupuncture signal initiation.

## Background

Microdialysis has been widely used in life sciences. Compared to other sampling techniques, microdialysis has distinctive advantages. The microdialysis probe can be placed directly, without causing much damage, into the target organs or tissues such as skin [1, 5, 16], muscle [10, 17], brain [6, 9, 11], blood vessels [15], and other areas. The sampling process can be continuous for hours or even days without major changes in the microenvironment. Moreover, macromolecules, such as proteins, are excluded from the semipermeable membrane so that the samples can be directly used for subsequent analysis without a cumbersome purifying procedure [18].

Due to its ability for high temporal resolution recording of ion channel currents from cells or cell-free membrane patches, the patch clamp technique has become the primary method for studying cell signal transduction mechanisms. Different types of ion channels have been studied, such as voltage-gated K+ channels [2], ligand-gated 5-HT3 (5-hydroxytryptamine) receptor channels [12], and mechanosensitive TRPV4 (transient receptor potential vanilloid) channels [19]. In vivo experiments have also been carried out though there are many limitations because of the difficulties associated with in vivo studies [13].

Acupuncture has been used to treat numerous diseases. As the starting point of acupuncture, acupoints play an important role in analgesia. Physical stimuli is changed to biological signals, after a series of complicated process, it reaches the target organs or tissues [23]. Having a better understanding of initiation of acupuncture will undoubtedly do us a favor to know the whole mechanism. Several studies indicate that there are more mast cells around acupoints than around non-acupoints [14], and these cells participate in the mechanism of acupuncture analgesia [21]. During acupuncture, mast cells are activated to degranulate. Several mediators released by mast cells, such as histamine and adenosine activate sensory nerve fibers [3]. The interaction of nerves, mast cells and chemicals around acupoints participates in the initial regulation of acupuncture [22]. Among the mediators, ATP activates P2X3 receptors located on sensory nerve endings [4, 20]. When ATP is degraded to adenosine, it also induces analgesia through adenosine A1 receptors [7]. If we can detect the dynamic changes of ATP concentration in acupoints during acupuncture, we can better understand the underlying mechanism of acupuncture. However, current sampling and analyzing techniques (e.g., microdialysis and HPLC, [7]) do not have high enough temporal resolution. Thus, we developed a new detection technique combining microdialysis with patch clamp to make it possible to analyze the substances in acupoints in real time.

## Results

### Different concentrations of ATP can be detected by the system in real time in vitro

**Fig. 1:**
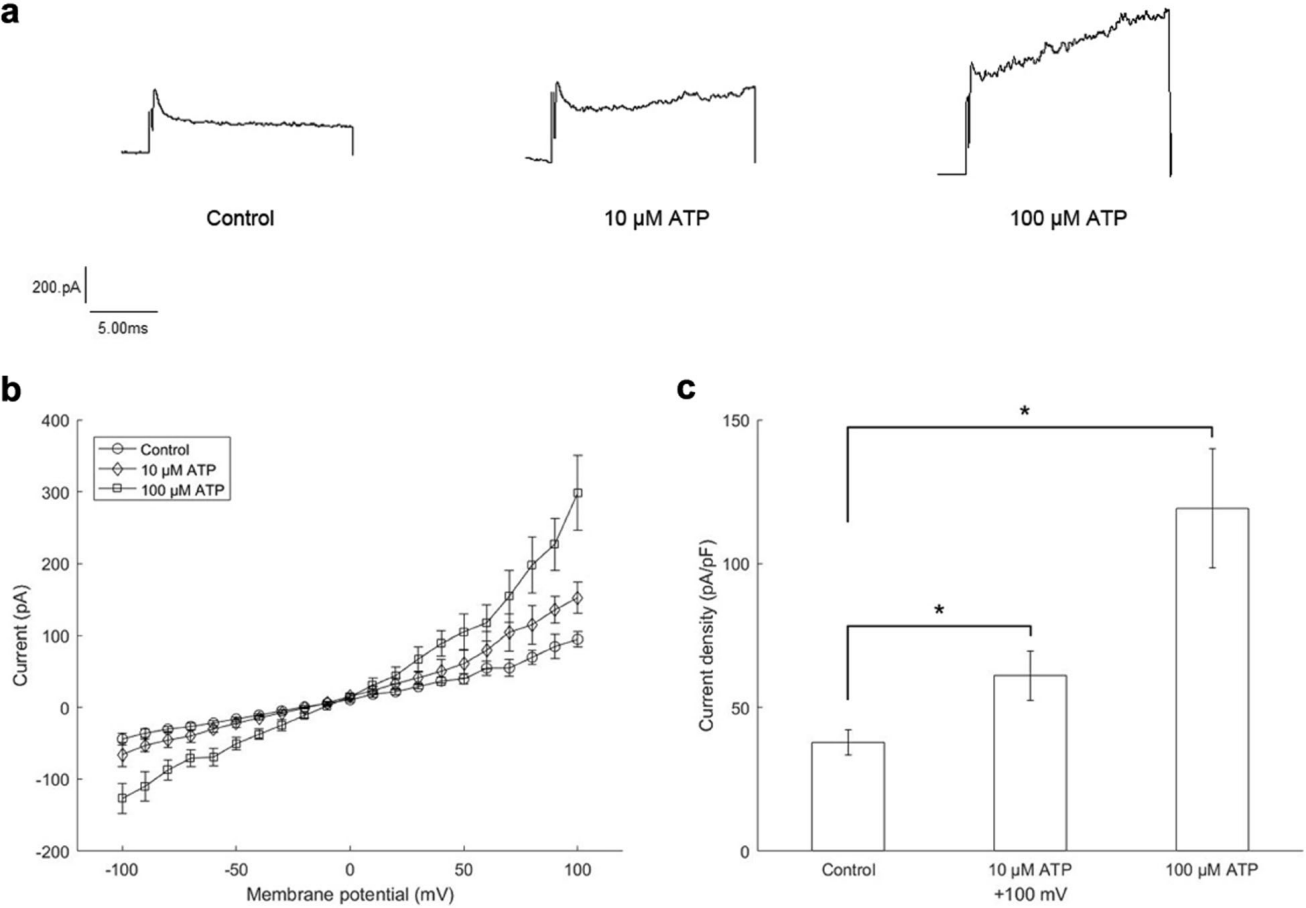
Membrane currents recorded with 293TX cells. **a** Actual current traces. **b** Summarized data of current-voltage relations. **c** Differences in current densities at + 100 mV. Current densities with control, 10 μM ATP and 100 μM ATP were separately 37.9 ± 4.4 pA/pF, 61.0 ± 8.7 pA/pF and 119.3 ± 20.8 pA/pF. * *P* < 0.05 vs control (*n* = 8)

First, we performed in vitro experiments with different concentrations of ATP standard solutions (10 and 100 μM). 293 T cells overexpressing P2X3 receptors (293TX for short) were used for the patch clamp recording. Membrane currents were elicited by 15 ms test pulses from − 100 mV to + 100 mV at a step of 10 mV with a holding potential of − 60 mV. During the entire process, currents were recorded continuously. The microdialysis probe was separately put into Ringer’s solution, or 10 μM and 100 μM ATP standard solutions. As ATP was dialyzed into the chamber, the membrane currents changed. The actual current traces are shown in Fig. 1a. Summarized data of current-voltage relations under different conditions are shown in Fig. 1b. The difference in current densities at + 100 mV is shown in Fig. 1c. Current densities with 10 μM ATP were 61.0 ± 8.7 pA/pF (*P* < 0.05), and current densities with 100 μM ATP were 119.3 ± 20.8 pA/pF (*P* < 0.05), significantly different from the control (37.9 ± 4.4 pA/pF). These results indicate that the system can be effectively used for the measurements in vitro.

### Changes in cell membrane currents are induced by ATP action on P2X3 receptors

**Fig. 2:**
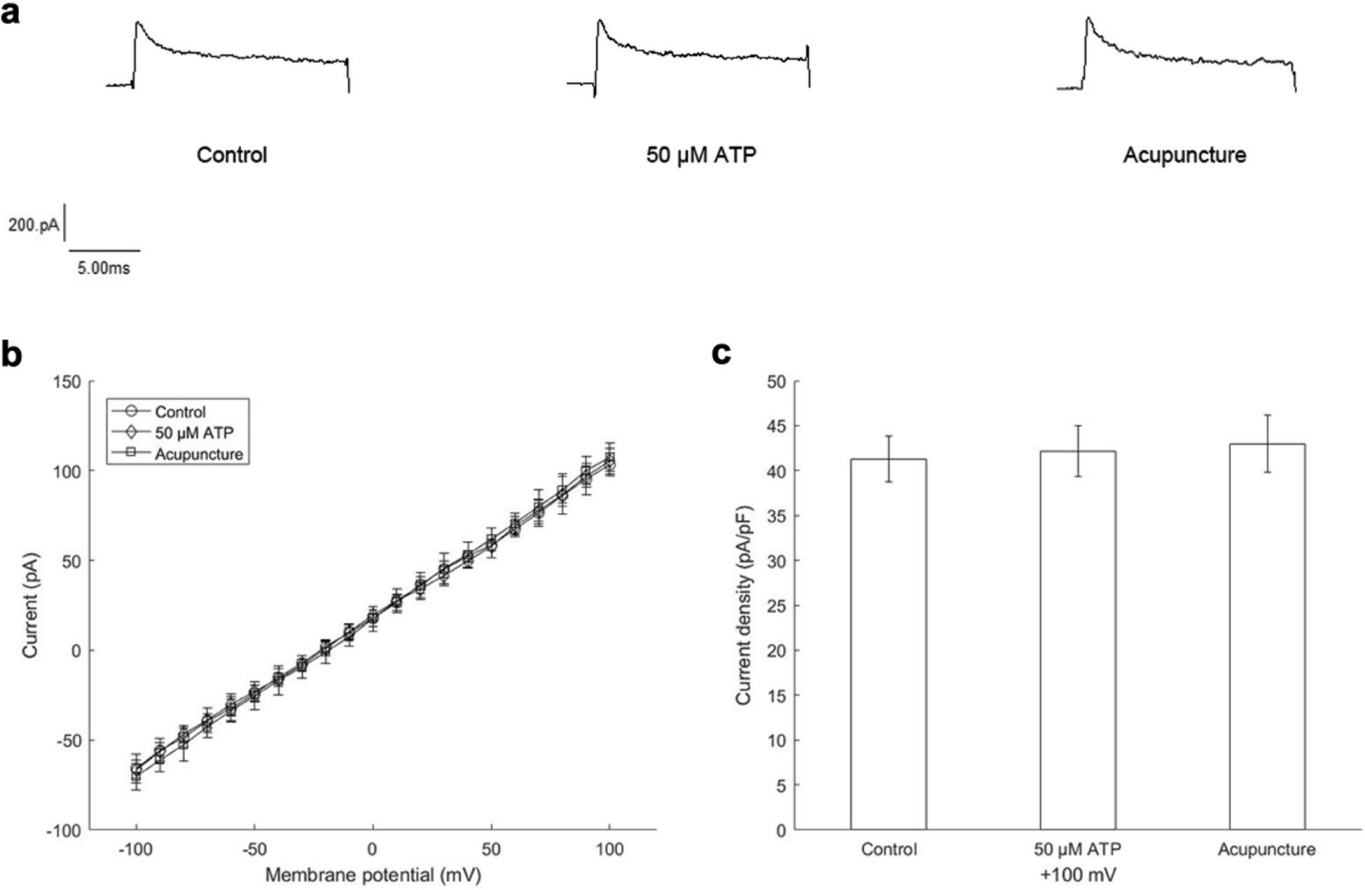
Membrane currents recorded with 293 T cells. **a** Actual current traces. **b** Summarized data of current-voltage relations. **c** Differences in current densities at + 100 mV. Current densities with control, 50 μM ATP and acupuncture were separately 41.3 ± 2.5 pA/pF, 42.2 ± 2.8 pA/pF and 43.0 ± 3.1 pA/pF. * *P* < 0.05 vs control (*n* = 8)

The cells played an important role in the stability of the experimental system. To ascertain that the changes in the membrane currents were only induced through P2X3 receptors activated by ATP, we performed in vivo experiments using 293 T cells without P2X3 receptors for comparison. Test pulses were the same as above. The microdialysis probe was separately put into Ringer’s solution, 50 μM ATP standard solution or the Zusanli acupoint (ST 36) of SD rats. After the probe was implanted into the acupoint for a period of time, we carried out acupuncture. As shown in Fig. 2a, the actual current traces did not show apparent changes. Summarized data were not significantly different (Fig. 2b,c). Current densities at + 100 mV were 41.3 ± 2.5 pA/pF (control), 42.2 ± 2.8 pA/pF (50 μM ATP) or 43.0 ± 3.1 pA/pF (acupuncture) separately. Therefore, we conclude that current changes are induced by ATP action on P2X3 receptors.

### Changes in the concentration of ATP in acupoints during acupuncture can be reflected by the system in real time

**Fig. 3:**
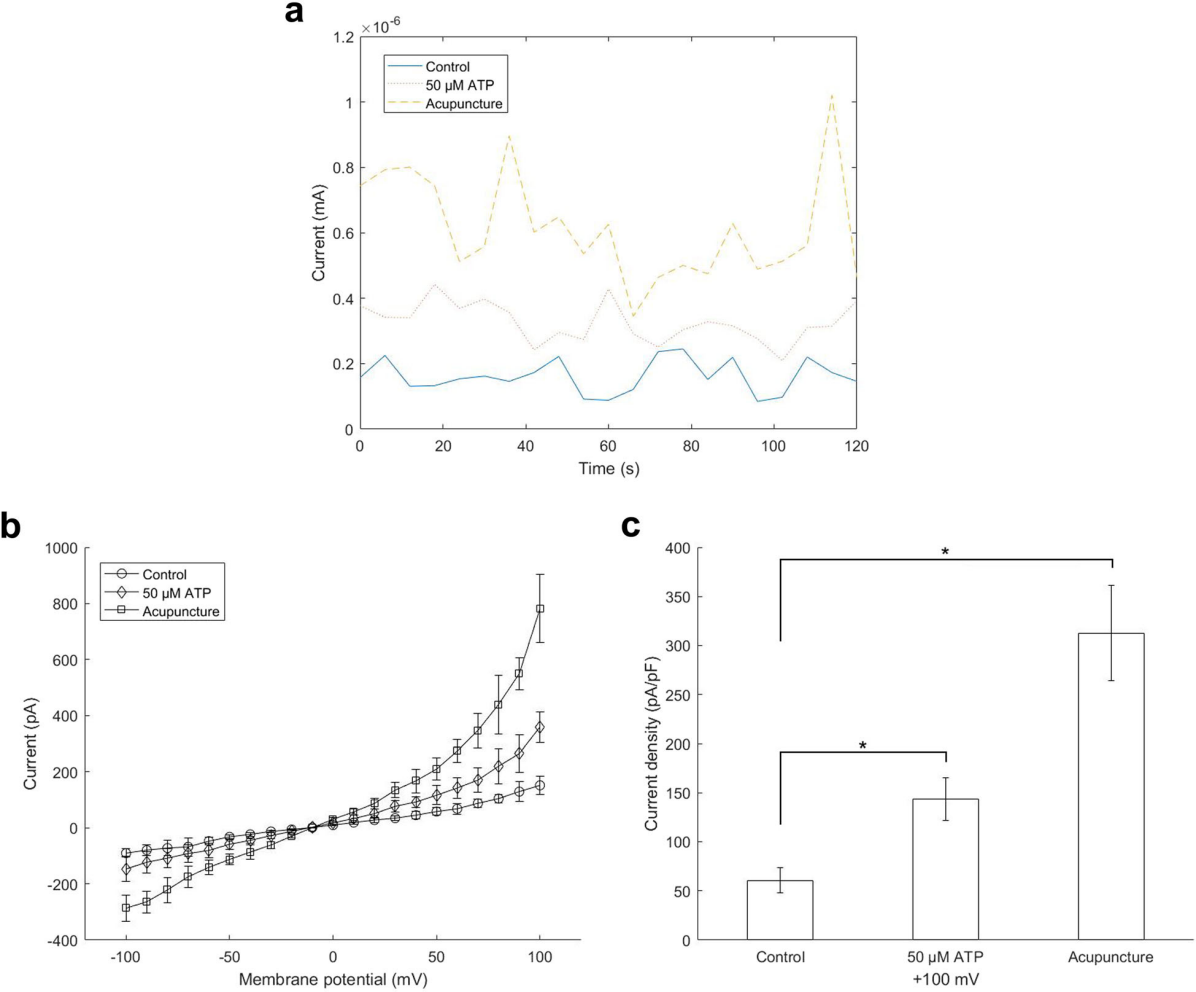
Membrane currents recorded with 293TX cells. **a** Current-time relationship at + 100 mV. **b** Summarized data of current-voltage relations. **c** Differences in current densities at + 100 mV. Current densities with control, 50 μM ATP and acupuncture were separately 60.7 ± 13.0 pA/pF, 143.7 ± 21.8 pA/pF and 312.6 ± 48.8 pA/pF. * *P* < 0.05 vs control (*n* = 8)

Next, we performed in vivo experiments using 293TX cells for patch clamps. The current-time relationship at + 100 mV is shown in Fig. 3a. When the probe was transferred to an ATP standard solution, the current increased. When the probe was implanted into the acupoint under acupuncture for a period of time, the current also exhibited a significant increase.

Summarized data of current-voltage relations are shown in Fig. 3b. Current densities with 50 μM ATP or acupuncture were 143.7 ± 21.8 pA/pF (*P* < 0.05) or 312.6 ± 48.8 pA/pF (*P* < 0.05), respectively. Both had significant differences compared to the control (60.7 ± 13.0 pA/pF) (Fig. 3c).

**Fig. 4:**
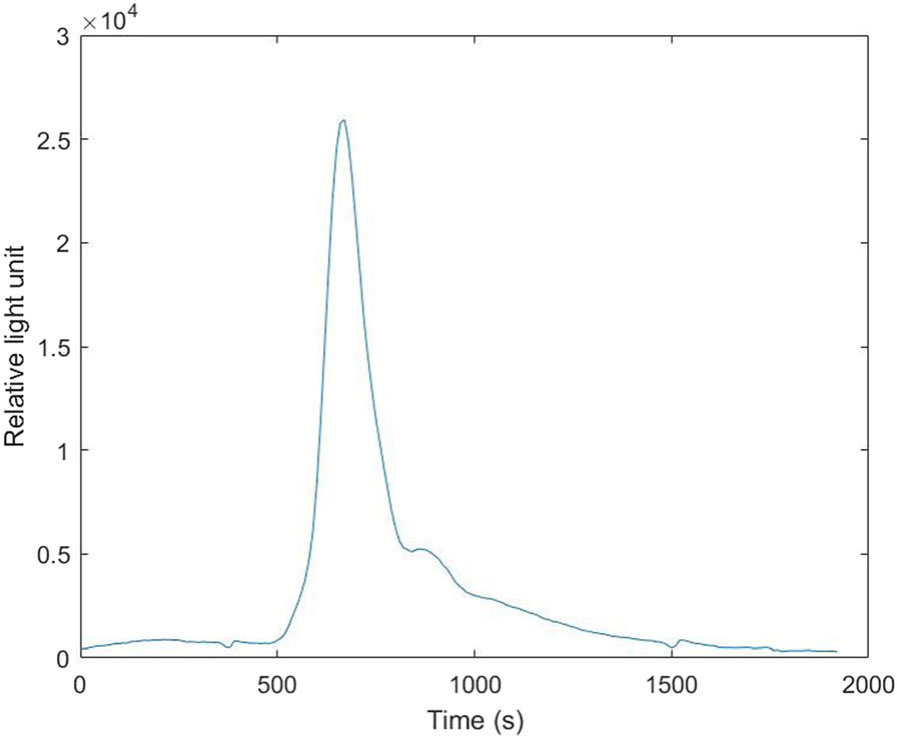
Relative light unit-time relationship of the fluorescence detection experiment

We also performed an ATP fluorescence detection experiment to further confirm the feasibility of our system. We changed the patch clamp system to a fluorescence detection system. When ATP samples were dialyzed into the luminometer, they reacted with luciferin and emitted light. The intensity of the light could be detected by the luminometer and the numbers of relative light unit were given out, which could reflect the concentration of ATP. The result is shown in Fig. 4. When we carried out acupuncture in the rats, the relative light units increased, which meant that the concentration of ATP increased. This result was consistent with our patch clamp results.

## Discussion

ATP plays an important role in the starting process of acupuncture. Current studies indicate that acupuncture can prompt the mast cells around acupoints to degranulate and release ATP, which activates P2X3 receptors of sensory nerve endings. Then, acupuncture signals are transduced, and acupuncture efficacy is achieved [4, 20]. We may have a better understanding of the underlying mechanism of acupuncture if we can determine the changes in ATP during acupuncture in real time. This new technique combining microdialysis with a patch clamp makes it possible to detect ATP electronically and in real time. We used microdialysis to extract ATP from the acupoints of the rats during acupuncture and delivered it to 293 T cells overexpressing P2X3 receptors for patch clamp recording. The results showed that changes in the concentration of ATP could be reflected by cell membrane currents in real time. Compared to other detecting techniques, our technique has a higher temporal resolution. This new technique also has the advantages of a small sampling volume and causing little damage to tissues.

However, there are still some challenges we must take into consideration. First, the distance between the microdialysis probe and the acupuncture needle can previously influence the concentration of ATP dialyzed out. However, both of them are under the skin, which means it’s difficult to keep them at the same distance during acupuncture. Maybe a visualized method is needed in the future to keep the results stable. Second, the results we obtain just qualitatively reflect the changes of concentration of ATP. Since the recovery of the microdialysis tube is affected by several factors, we haven’t make a calibration to get the quantitative results yet. It has to be finished in further experiments.

## Conclusions

By choosing different cells expressing specific receptors, we can detect different types of mediators such as histamine and 5-HT online and in real time using our technique. As we know, acupuncture signals are conducted to central nervous system firstly, then to target organs or tissues. Apart from local acupoints, our technique can also be used in brain research or other research areas. We believe that this technique will have a broader application in the future.

## Methods

### System construction

**Fig. 5:**
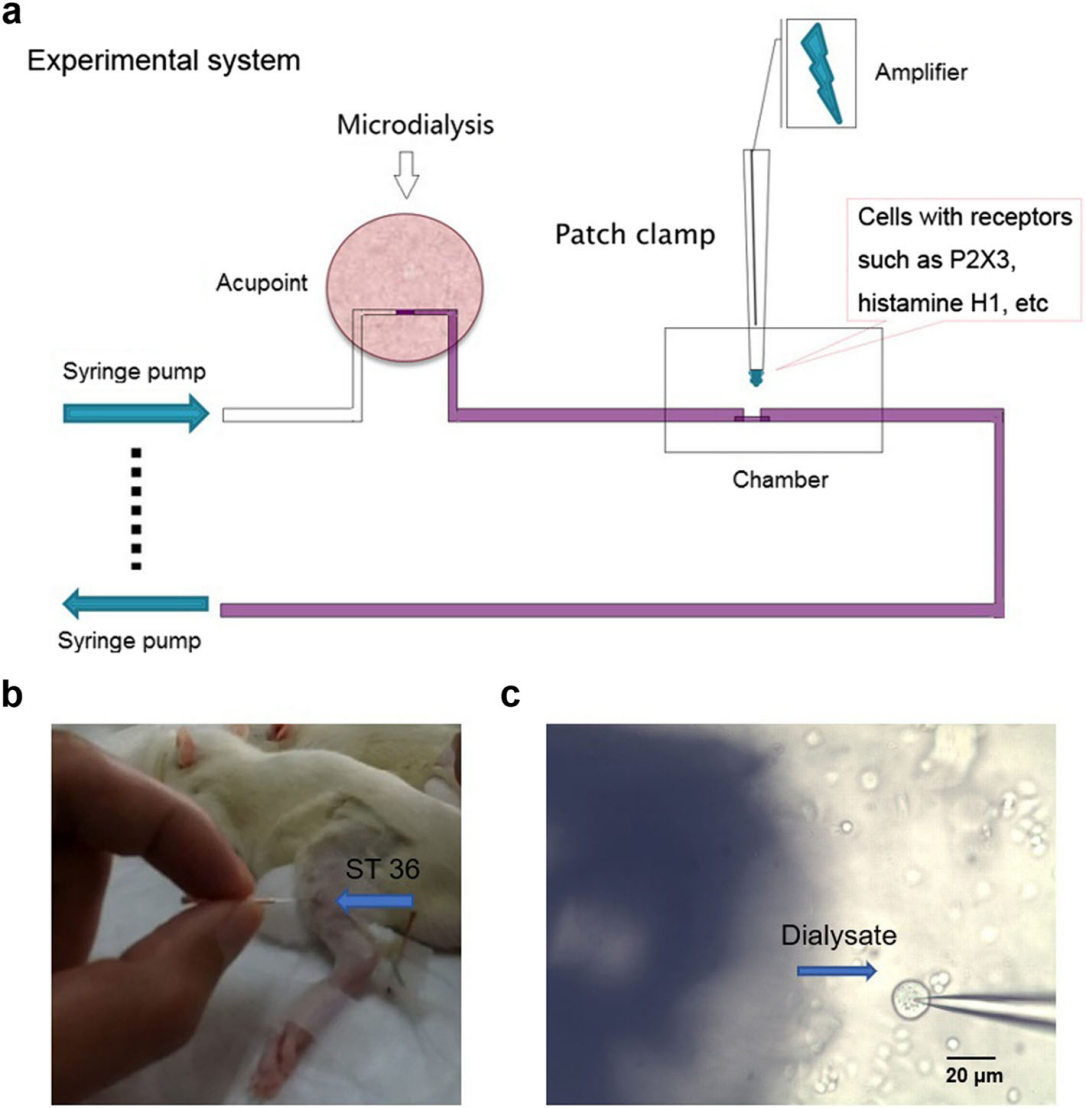
Diagram of the experimental system**. a** Illustration of the whole system. Mediators from the acupoints are pumped into the chamber through the microdialysis tube. Cells expressing different receptors are used to record the currents. **b** A microdialysis tube was implanted into the rat’s Zusanli acupoint (ST 36). **c** The cell was patch-clamped by the pipette. Shadow on the left was the tube through which the perfusate was pumped into the chamber

A special chamber was made to connect the microdialysis system with the patch clamp system, as shown in Fig. 5. Samples from animals were dialyzed through the microdialysis tube. Then, the samples were pumped into the chamber with the cells overexpressing specific receptors and the currents were recorded from these cells. The changes in ATP concentration of the samples can be reflected by those of cell membrane currents.

### Cell preparations

**Fig. 6:**
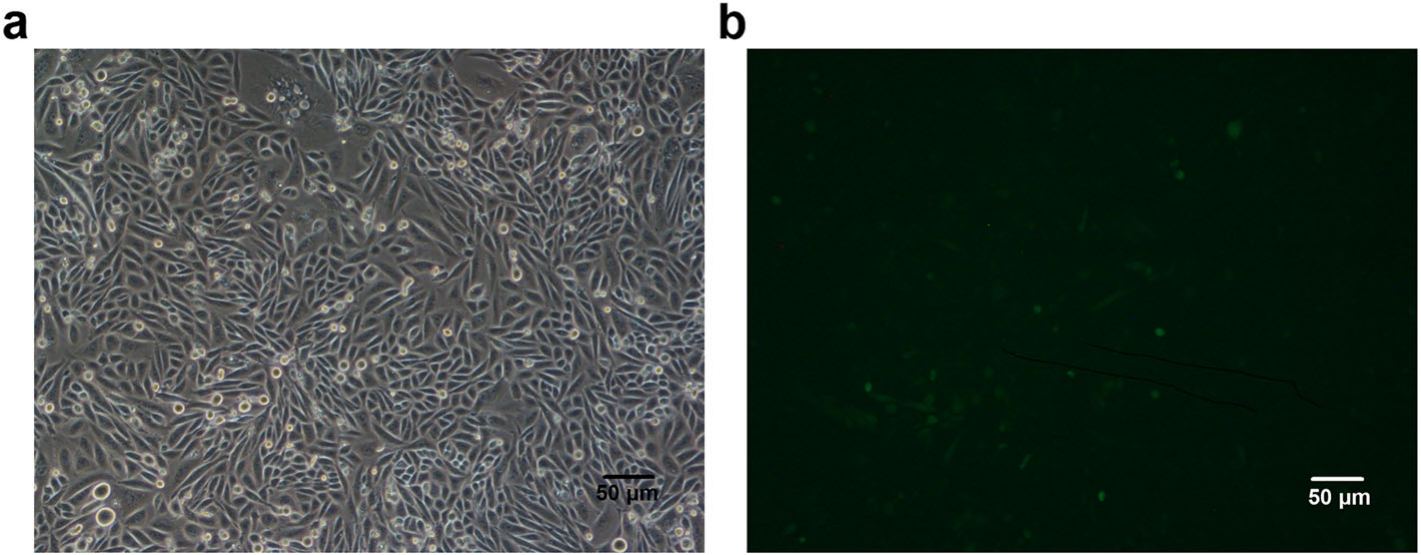
293 T cells observed under the microscope**. a** Cells under the bright field. **b** Cells under the fluorescence microscope. Those with green fluorescence were successfully transfected and overexpressing P2X3 receptors

We chose 293 T cells to carry out patch clamp experiments, since 293 T cells rarely express endogenous receptors required by extracellular ligands and are relatively easy to be transfected. 293 T cells were provided by the Cell bank of the Chinese Academy of Sciences (Shanghai, China). Then, they were stably transfected with P2X3 receptors (Fig. 6) by the Shanghai Research Center of the Southern model organisms. Cells were cultured with DMEM (Gibco, Invitrogen, Grand Island, NY, USA) supplemented with 10% fetal bovine serum (Gibco, Invitrogen, Australia) and 1% penicillin and streptomycin (Gibco, Invitrogen, Grand Island, NY, USA) in a 95% humidity-controlled incubator with 5% CO_2_ at 37 °C. Before the experiment, the cells were washed with phosphate buffer saline (Gibco, Invitrogen, Grand Island, NY, USA) for 0.5 min. Then, the cells were digested with trypsin-EDTA (Gibco, Invitrogen, Grand Island, NY, USA) for 5 min and transferred onto a small square slide until they adhered and it was convenient to put them into the chamber.

### Animal preparations

All animal experiments have followed ARRIVE guidelines. SD rats were provided by the Shanghai laboratory animal center of Chinese Academy of Sciences. Before the experiment, the rats were anesthetized with chloral hydrate (0.4 ml/100 g). Fur around the Zusanli acupoint (ST 36) was shaved to expose the skin. A plastic annular tube with a larger diameter was inserted into the skin so that the microdialysis tube could be easily implanted into the issue. After this manipulation, animals were allowed to rest for 30 min.

### Experimental solutions

The composition of the normal bath solution was (mM): 140 NaCl, 5 KCl, 1 CaCl_2_, 1 MgCl_2_, 10 D-sorbitol, and 10 HEPES and was titrated to pH 7.4 with NaOH. The pipette solution had the following composition (mM): 140 CsCl, 1 CaCl_2_, 1 MgCl_2_, 5 EGTA, and 10 HEPES and was titrated to pH 7.2 with CsOH. Ringer’s solution was used as the perfusate through the microdialysis tube. All solutions were stored at 4 °C until use.

### Microdialysis and whole-cell current recordings

During the entire recording process, a microdialysis tube (BASi Instruments, USA) was placed into Ringer’s solution, ATP standard solutions or SD rats. When the tube was implanted into the SD rats, acupuncture was carried out at the Zusanli acupoint (ST 36). The acupuncture needle (Hwato, China) was Ø 0.35 X 25 mm.

Whole-cell membrane currents were recorded by the patch-clamp method [8]. Cells were placed into the chamber attached to an inverted microscope (Nikon, Japan) and superfused with the bath solution by gravity at a rate of 1 ml/min. The temperature of the external solution was at room temperature (25 °C). Patch pipettes were made from glass capillaries with a diameter of 1,5 mm (WPI, USA) using a horizontal microelectrode puller (model P^− 97^, Sutter Instrument, USA). The patch pipettes were filled with the pipette solution, and their resistance was 2–5 MΩ when they entered the bath solution. The glass pipette was held by an electrode probe connected to a patch-clamp amplifier (model EPC-10, HEKA, Germany). The probe was controlled by an electronic micromanipulator (model MPC-2000, Sutter Instrument, USA). Command pulse signals were generated using PATCHMASTER software (KEKA, Germany). Current data were also acquired by PATCHMASTER and stored on the hard drive disk of a computer (Samsung, South Korea). Recording signals were filtered at 2 kHz bandwidth, and series resistance was not compensated.

### Statistics

Statistical analyses were performed with one-way analysis of variance (ANOVA) followed by Student’s t-test for paired values. Changes were considered significant at *P* < 0.05. Data were expressed as the mean ± S.E.M.

## Data Availability

The datasets used and/or analysed during the current study are available from the corresponding author on reasonable request.

## Abbreviations

293TX: 293 T cells overexpressing P2X3 receptors
5-HT: 5-hydroxytryptamine
ATP: Adenosine triphosphate
SD: Sprague Dawley
TRPV: Transient receptor potential vanilloid
